# Collectanea

**Published:** 1831-01

**Authors:** 


### COLLECTANEA.

Floriferis ut apes in saltibus omnia libant,
Omnia nos, itidem, depascimur aurea dicta.

### PHYSIOLOGY.

*Vaccine Virus.* Dr. Robert, physician to the Lazaretto at Marseilles, is of opinion, that cow's milk communicates to the variolous virus the properties of vaccine virus. Thirteen experiments have been made by him, and communicated to the Institute of France, which seem to show that the virus of variola, and varioloid combined with cow's milk, when used for inoculation, produces only a local eruption, similar to that of vaccina, proving, he thinks, that the vaccine disease originated in the accidental transmission of the variolous virus from man to the cow's teat, which is the cause of its mildness.—*Journal de Chimie Med.*

### PATHOLOGY.

*Hernia of the Stomach, with enormous Enlargement of that Organ.* This case was communicated to the Royal Academy of Medicine by M. Yvan. The subject was a soldier, who for some years had had a scrotal hernia, which could be easily reduced by taxis, but could not be kept up: it gradually increased in size, and a month previously to his death repeated vomiting took place, which could not be arrested, although there was no strangulation, and the patient died. On examination, the abdominal ring was found to be enormously distended, measuring eighteen inches in circumference; the hernial sac contained the inferior third of the stomach, the greater omentum, the small intestines, and the large intestine, except the iliac portion of the colon, the cœcum, and the rectum. The stomach, situated parallel to the axis of the body, was of an enormous volume, and seemed divided by a circular depression into two portions; the one situated in the abdomen, the other in the hernial sac. The length of the great curvature of the stomach was three feet, that of the lesser curvature, eighteen inches; its circumference at its largest part twenty inches, and contained nearly eleven pints of fluid. Longitudinal and circular muscular fibres were observed on the surface of this organ. The stomach is in the museum of the Academy.—*Archives Générales.*

***Poisoning by Cantharides in Powder, followed by the Expulsion of the Mucous Membrane of the Œsophagus.*** By M. Rouquariol. A shoemaker, aged forty-six, poisoned himself by swallowing cantharides. Three hours afterwards, when Dr. R. saw him, he was affected with the following symptoms: burning heat of the mouth, throat, and stomach; pains in the kidneys and bladder, incessant but ineffectual desire to void urine; mouth excoriated; ptyalism; nausea, vomiting; tongue trembling; convulsive movements; priapism; contracted and frequent pulse; in the matter vomited, portions of mucous membrane and of cantharides could be recognized. Dr. R. administered half a pint of olive oil, and soon afterwards twenty grains of ipecacuanha, which induced vomiting. He gave the olive oil, because it dissolves the active principle of the cantharides, is an article not easily digested, and is much less readily assimilated than milk or any other similar substance. The quantity of the poison taken had evidently been large, judging from the quantity seen in thc matters vomited. The patient was afterwards forced to swallow two pints of olive oil, and half an hour afterwards two ounces of castor oil: these produced copious evacuations from the bowels, containing a large quantity of cantharides; the doses of olive oil and castor oil were repeated, and with the same results.

Evening. Convulsive movements; continual priapism; bloody urine; febrile heat of the abdomen, frequent contracted pulse; subsultus tendinum; pains in the throat, stomach, kidneys, and bladder continue. Ordered olive oil by the mouth and anus, ten leeches to the neck, twenty to the epigastrium, and twenty to the perineum; afterwards patient to be placed in a tepid bath for three quarters of an hour.

The next day there was a slight abatement of the symptoms, except the pain in the bladder; thirty leeches were applied to this part, and flaxseed tea for drink, emollient enemata, and a tepid bath were ordered.

Evening. More calm; but at eleven o'clock vomiting recurred, and the entire mucous membrane of the œsophagus was discharged.

June 10th. Better; same prescription as last night, except the leeches; deglutition extremely painful.

The patient from this time gradually improved, and on the 22d of June he took some solid food. He now enjoys perfect health, better than he did before his poisoning, when he was affected with a chronic duodeno-hepatitis, which at times became acute.

Not the least interesting and remarkable circumstance in this case is the expulsion of the mucous membrane of the œsophagus, which took place with much pain, accompanied with the discharge of blood. Its middle portion, for an inch and a half, was entire, and formed a tube, the extremities of which were ragged. Some particles of cantharides were adhering to its internal surface, on its external surface, blood-vessels running longitudinally, as also folds or pleats in the same direction were perceptible. One of these vessels was so large, that blood was pressed out of it after puncturing it with a lancet; this membrane could not, of course, have been an adventitious one.—*Annales de la Médecine Physiologique.*

---

SURGERY.

*A Case of Aneurism of the External Iliac Artery, in which a Ligature was applied to the Common Iliac Artery.* By PHILIP CRAMPTON, M.D. F.R.S. Surgeon General to the Forces in Ireland, and Surgeon in Ordinary to the King.

To Mr. Mott, of New York, is due the merit of having been the first to suggest, and the first to effect, the ligature of the common iliac artery. The following case affords a second instance in which a similar operation has been performed. To the practical surgeon, it will lose nothing of its interest, either because it is not the first, or because it has not been successful. In fact, paradoxical as it may appear, the progress of surgery is more likely to be advanced by a faithful account of unsuccessful than of successful cases. In the successful cases nature performs her work in secret, and often leaves us at a loss to determine whether the success which we witness is to be attributed to the efforts of art, or to the unassisted resources of nature. In the unsuccessful cases, on the contrary, we have usually an opportunity of examining after death the effects of our operations; we see the results of those efforts which, in concurence with or in opposition to ours, nature makes for her relief, and are thus enabled to discriminate between the means which are likely to advance, and those which are likely to retard her work. I am further induced to submit this case to the consideration of the surgical profession, because in the operation I adopted a proceeding different from Mr. Mott's, and one which renders the tying of the common iliac artery, or even of the abdominal aorta itself, (so far as the operation itself is concerned,) comparatively easy and safe.

CASE. Private P. M'Gowan, 8th Foot, aged thirty, well formed, and hitherto healthy, was admitted into the General Hospital, Phœnix Park, Dublin, on the 8th of July, 1828. There was a pulsating tumor extending from about three inches below the crural arch on the right side, to within about three inches of the umbilicus. The tumor was divided by a furrow in the line of Poupart's ligament, into two portions.

The lower, or femoral portion, was scarcely compressible, and, although throbbing strongly, did not communicate to the hand the peculiar "aneurismal thrill." The upper, or abdominal portion, which formed an obvious swelling in the right hypogastric region, was soft and compressible, as if filled with fluid blood, and at a particular point, about three inches above the crural arch, the aneurismal thrill, (which is caused by the jet of blood from the ruptured artery passing into the fluid blood of the aneurismal sac,) was unusually distinct. There was also a pulsating and compressible tumor, about the size of a pullet's egg, in the right ham.

The man complained of great pain in the thigh and leg, which prevented his walking, and disturbed his rest: pulse 110, full and throbbing, tongue white, but little appetite. He attributes the disease to a fall which he got in wrestling about nine months previously. He felt but little pain at the time, but two days afterwards he observed a pulsating tumor, about the size of a walnut, at what he terms "the rim of his belly." He continued to do his duty until the 20th of May, when the tumor becoming much larger and more painful, he reported himself to the regimental surgeon. During the last fortnight, the swelling has increased rapidly, and the pain is so severe, that he says he is satisfied to submit to any operation which may afford a chance of relief. From the 8th to the 17th of July, he was bled five times. He was put on fever diet, purged daily, and took from twenty to thirty drops of tincture of digitalis, three times a day. The blood, which for the first four bleedings was highly buffed and cupped, presented on the 5th a natural appearance, the pain and fever had abated, and on the 18th of July he was considered to be in a favorable state for the operation.

The operation was accordingly performed at two o'clock P.M., on the 18th of July, in the presence of Professors Colles, Macartney, aud Wilmot, Mr. Stringer, (the surgeon to the hospital,) Dr. Ramsay, of Dundee, and several other professional gentlemen.*

*The Operation.* The first incision commenced at the anterior extremity of the last false rib, proceeding directly downwards to the os ilium, it followed the line of the crista ilii, keeping a very little within its inner margin, until it terminated at the superior anterior spinous process of that bone; the incision was therefore chiefly curvilinear, the concavity looking towards the navel. The abdominal muscles were then divided to the extent of about an inch, close to the superior anterior spinous process, down to the peritoneum: into this wound the forefinger of the left hand was introduced, and passed slowly and cautiously along the line of the crista ilii, separating the peritoneum from the fascia iliaca, the peritoneum touching the fore part, and the fascia iliaca the back part of the finger. A

* I think it may not be amiss to observe, in this place, that previously to my performing the operation on M'Gowan, I was enabled, by the kindness of the gentlemen who were conducting summer courses of anatomy, to perform preparatory dissections and operations on seven dead subjects. Two of the operations were performed at the Richmond Anatomical School, on the morning of the 18th, and on each trial, even to the two last, I found that I performed the operation in a manner much more satisfactory to myself than any of the preceding ones. I mention this not only as a matter worthy of the consideration of those who would limit the opportunity of the student of surgery to the dissection of two, or at the utmost four subjects, but as affording a proof, if indeed proof be wanting, that operative surgery cannot be usefully cultivated without almost unlimited means of practising dissection, and that, as it has been well expressed, "We must choose between mangling the living or the dead."

probe-pointed bistoury was now passed along the finger to its extremity, and by raising the heel of the knife, while its point rested firmly on the end of the finger, as on a fulcrum, the abdominal muscles were separated from their attachments to the crista ilii by a single stroke. By repeating this manœuvre, the wound was prolonged until sufficient room was obtained to pass down the hand between the peritoneum and the fascia iliaca. Detaching the very slight connexions which these parts have with each other, I was able to raise up the peritoneal sac, with its contained intestines, on the palm of my hand, from the psoas magnus and iliacus internus muscles, and thus obtain a distinct view of all the important parts beneath; and assuredly a more striking view has seldom been presented to the eye of the surgeon; the parts were unobscured by a single drop of blood; there lay the great iliac artery, nearly as large as my finger, beating awfully at the rate of 120 in a minute, its yellowish white coat contrasting strongly with the dark blue of the iliac vein which lay beside it, and seemed nearly double its size; the ureter, in its course to the bladder, lay like a white tape across the artery, but, in the process of separating the peritoneum, it was raised from it with that membrane to which it remained attached. The fulness of the iliac vein seemed to vary from time to time, now appearing to rise above the level of the artery, and now to subside below it. Nothing could be more easy than to pass a ligature round an artery so situated. The forefinger of the left hand was passed under the artery, which, with a little management, was easily separated from the vein; and on the finger, (which served as a guide,) a common-eyed probe furnished with a ligature of moistened catgut was passed under the vessel. A surgeon's knot was made in the ligature, and the noose gradually closed, until Mr. Colles, who held his hand pressed upon the tumor, announced that "all pulsation had ceased!" A second knot was then made, and one end of the ligature cut off short. On examining the vessel after it had been tied, it was found to be full, and throbbing above the ligature, but empty and motionless below it. The external wound was united by three or four points of suture, and supported by long straps of adhesive plaster. The operation was completed in twenty-two minutes: the patient, who was a firm-minded man, made no complaint during the operation, not even when the ligature was closed upon the artery. The tumor, immediately after the operation, was diminished nearly one third, the diminution being confined to the abdominal portion: ten minutes after the operation, the pulse was ninety-six; at seven P.M. Mr. Stringer, finding the pulse full and bounding, took twenty ounces of blood from the arm; at ten P.M. I found him tranquil, no pain, pulse eighty-eight, the limb with the exception of the toes, warm: saphena vein full; additional flannel was wrapped round the foot.

Saturday 19th. Had some sleep; no sense of throbbing, pulse eighty-eight, toes warm, but not so warm as those of the left foot; no pain of the abdomen, even on pressure; bowels not open. Ordered an ounce of castor oil, and an enema if necessary.

Sunday, 20th. An uneasy night from pain and rumbling in the bowels; no stool, temperature of both limbs equal: five grains of calomel, three hours afterwards an ounce of castor oil, and two drachms of oil of turpentine, opened the bowels at four o'clock, and removed all uneasiness from the abdomen. It was now, just fifty hours after the operation, first observed by Mr. Corr, (one of the house pupils,) that there was an obscure pulsation in the tumor.

Monday, 21st. There is decidedly a pulsation in the whole tumor, obvious both to the touch and to the eye, but there is no "thrill," as if the contents of the sac were fluid. No pulsation in the femoral artery below the tumor, and none whatever in the aneurism at the ham, which is reduced to half its original bulk. Pulse eighty-eight, skin cool; no pain; temperature, as ascertained by repeated trials with the thermometer, at the groin 98°, at the hams 97°, at the ancles 94°, at the ball of the left great toe 87°, at the right 88°.

Tuesday, 22d. Pulsation still evident, accompanied (in the opinion of some of the observers) with an obscure thrill; tumor not increased; no pain; pulse eighty-six; no pulsation in the ham.

23d. No change.

24th. Little or no change, unless that the pulsation in the abdominal portion of the tumor is perhaps more distinct. Ordered to be bled, in the erect position, ad deliquium; tincture of digitalis, twenty drops every third hour; fever diet.

The object of this treatment was to diminish the force of the circulation through the tumor, in the hope that its contents might coagulate. It was plain, that from whatever source the blood was derived, it was flowing into the aneurismal sac, but with diminished force.

25th, (7th day.) Countenance pale, pulsation more distinct, the thrill quite perceptible, no pulsation in the femoral artery below the crural arch; blood drawn yesterday buffed, and cupped; twelve ounces of blood taken from the arm; digitalis increased to thirty drops, three times a day; diet as before.

26th, (8th day.) The ligature came away last night, but was lost in the bed; pulse as yesterday; on turning in the bed, he suddenly felt a severe pain in the thigh and knee "as if" (to use his own expression) "the knee was tearing off of him"; said "the fore part of the thigh was all numb." He cried out from pain: in ten minutes it subsided.

It now became a subject of great interest, to determine the cause of the return of the pulsation, after it had ceased for fifty hours. Has the blood returned by the free anastomosis between the internal iliacs? or has the catgut ligature become macerated, and given way? The pulsation is stronger than could be expected, if the tumor were supplied only by the collateral source of anastomosis; the prevalent opinion therefore was, that the ligature had given way.

27th. Less pain in the knee and thigh; in other respects as yesterday.

28th. As yesterday, but complains less; asks for nourishment: the wound is quite healed, with the exception of about an inch, in the middle of this line, the hole through which the ligature escaped is apparent. Pulsation in the tumor nearly as strong as before the operation; yet the whole bulk of the tumor is reduced by at least a third.

At six P.M., while he was sitting up in his bed, taking some gruel, the blood suddenly gushed from the wound, and deluged the bed. He fell backwards, and expired in less than a minute. The body was examined at one o'clock on the following day, in the presence of Messrs. Colles, Wilmot, Cusack, Stringer, Porter, and several other professional gentlemen.

*Dissection.* The intestines being removed, the peritoneum raised, and the great abdominal vessels laid bare, the common iliac artery, at about three fourths of an inch from the bifurcation of the aorta, was lost in an oblong tumor, about three fourths of an inch in diameter, and one and a half in length; the tumor terminated upon, but did not communicate with, the aneurismal sac. On cutting into the tumor, about half an ounce of greenish pus flowed from the wound, and discovered the artery, which appeared somewhat contracted at one part, and its coats deeply indented, but not cut through, marking the place where the ligature had been applied. On blowing into the iliac artery from above, bubbles of air escaped freely from the external wound from whence the blood had issued; water injected by a syringe escaped by the same passage: clearly establishing the important fact, that the ligature, which was of catgut, had been dissolved by the heat and moisture of the wound, and thrown off, before the obstruction of the artery, or the coagulation of the blood in the aneurismal sac, had been completed. It further appeared that the dissolution of the ligature had caused a small abscess to form in the place which it occupied. On slitting up the artery, the internal and middle coats were found to be completely divided in the whole circumference of the vessel, and small portions of lymph adhered to its internal surface. The popliteal aneurism was far advanced towards a cure; the contents of the sac were quite solid, and the tumor was reduced to about the size of a walnut: the artery, for six inches above the sac, was filled with a firm coagulum.*

The most important inferences to be drawn from this case are,

1st. That the operation of tying the common iliac artery, is not only a feasible, but (when performed in the manner described in this paper) is an exceedingly easy operation. The difficulties which Mr. Mott encountered, and which prolonged the operation "to nearly an hour,"† are clearly referable to the circumstance of his incision

* The parts are deposited in the museum at the Park street School.

† "The operation lasted rather less than an hour." Vide Dr. V. Mott, New York. "Successful Ligature of the Common Iliac Artery."

having been made too low. This in the first place brought him in contact with the aneurismal tumor, from which he was obliged, with great labour and considerable risk, to detach the peritoneum;* then he had the whole mass of the tumor between him and the artery which he was to tie: and lastly, he had the intestines pressing down upon him, and producing such a complication of difficulties, as I believe few men but himself could have encountered with success. All these difficulties however, might, I think, have been avoided, by getting at the artery from behind and above the tumor: in a word, by an incision which should begin where Mr. Mott's terminated.

2d. The question has often been proposed, "whether, under any possible circumstances, a surgeon would be justifiable in passing a ligature round the abdominal aorta?" Without venturing to give a decided opinion upon this subject, it may not be amiss to observe, that, in several instances, aneurisms of the abdominal aorta have undergone a spontaneous cure, in consequence of the obliteration of the artery above and below the tumor.

I have given, in the 2d volume of Dublin Hospital Reports, the history of a case of this kind; and the preparation illustrative of it, is now in the museum of Guy's Hospital, where it was deposited by Sir A. Cooper, to whom I transmittted it in the year 1819.

If such an operation should be determined upon, I have no doubt, that by a proceeding similar to that which I have described in this paper, a ligature could with great ease be passed round the abdominal aorta, without interfering with the cavity of the abdomen.—*Med.-Chir. Transactions.*

---

*Extirpation of a Cancerous Eye.* By Harvey Lindsly, M.D. of Washington, D.C. About the 1st of March, 1829, I was consulted by Michael Furman, respecting a disease of his right eye. He informed me that the complaint commenced about three years previously, in the form of a small white speck on the anterior surface of the eye; which, however, did not prevent his attention to his daily occupations, until some time in November, 1828, when it became painful, and began to enlarge with considerable rapidity.

He had consulted several physicians, and by their direction had employed various remedies without any beneficial effect, and in some instances with decided injury. Lunar caustic and astringent lotions were the principal articles made use of.

It should be mentioned that the patient was about fifty years of age, a carpenter by trade, and of intemperate habits.

As other remedial agents had failed, and as he was evidently getting worse, I deemed it my duty to extirpate the diseased mass, as affording the only chance of preserving the patient's life;

* "With the tumor beating furiously underneath, I now attempted to raise the peritoneum from it, which we found difficult and dangerous, as it was adherent to it in every direction." See "Successful Ligature," &c., by Dr. V. Mott, New York.

although owing to the length of time the disease had existed, and the irritable and debilitated state of his system, I placed but little dependence even on this last resort.

The disease consisted of two distinct masses: the one nearest the nose, of a dark red colour, and a soft and gelatinous consistence; the other of a dark purple, and cartilaginous hardness. Both together filled up entirely the cavity between the nose and eyebrows, so that in a front view the eyelids could not be seen.

On the 24th of March, I proceeded to extirpate the eye, in the presence of Drs. Sewall, Bullfinch, and Dunn, and several medical students. The mass was so large as to render it necessary to make an incision through the integuments at each angle, in order that the eyelids might be sufficiently retracted to proceed with the operation.

After completing the extraction in the usual manner, I filled up the cavity with lint, applying compresses, &c. The hemorrhage was considerable: fourteen to twenty ounces were probably lost, during and immediately after the operation, from the branches of the ophthalmic artery, though I did not find it necessary to apply any ligatures. A slight oozing followed, but ceased entirely in about twelve hours after the dressings were applied. Ordered two grains and a half of opium before leaving the patient.

25th. Has had a comfortable night, and slept soundly; pain very slight. Prescribed an aperient and light diet.

26th. Still doing well. I now placed the patient under the care of two intelligent students, with directions to change the dressings every morning, and keep the bowels open. Healthy granulations soon made their appearance, and in a month the cavity was entirely filled up, with little or no deformity. But notwithstanding the apparent success of the operation, I felt an anxious solicitude about the ultimate fate of my patient; fearing that the disease might return, and still prove fatal. My apprehensions were not without foundation, as, in about five months from the period of the operation, the disease returned. As he had removed from his former residence, I saw him but once during this time, but was informed that he died eight months afterwards.

Upon dissection of the extirpated mass, I found the humours wholly absorbed, and their situations occupied with a hard substance, of nearly the same colour as the external portion. Cysts, also, were interspersed, containing a substance equally hard, and of different colours, from a light ash to a dark purple or black. The sclerotic coat was a little thickened, but the optic nerve was of its natural size and colour.

This case is interesting, as it adds one more to the many proofs we already possess, of the importance of early extirpation in this formidable disease.

It is a fortunate circumstance that the distinction between cancer and fungous hæmatodes has been so clearly drawn, that no apology now exists for confounding them; this, at least, is the case, if the description of the disease in our systematic writers can be depended on. The diagnosis is important, inasmuch as it seems now generally conceded that extirpation of fungous hæmatodes, unless most cautiously performed, and in the very beginning of the complaint, is not only useless, but decidedly injurious; rendering the disease more rapid in its progress, and more inveterate in its character: whereas the removal of cancer has often been successful, even in a stage considerably advanced.—*American Journal of Med. Sciences.*

---

*Case in which a foreign Body remained ten Years in the Bronchia before causing Death.* M. Dupuytren relates the following case: —A robust young man, whilst playing with some children, amused them by throwing up a ten-sous piece, and catching it in his mouth; at last, during the moment of inspiration, the coin fell into the trachea. Violent painful cough, accompanied by a peculiar noise, immediately ensued, especially when the foreign body was, during expiration, thrown up towards the glottis; when it was not moved, as it sometimes happened, for several hours, respiration was but slightly affected. The patient being continually in hopes that the foreign body would be thrown up through the glottis, decidedly objected to an operation, and in this state continued for five years, during which time he was much inconvenienced by cough, suffocation, &c. After that period, however, the foreign body appeared to become fixed, and for some time the patient felt almost quite well. Symptoms of phthisis however gradually succeeded, and terminated his life ten years after the accident: the piece of money was found in a tuberculous excavation.

---

*Compound Tincture of Benzoin as a Remedy for Scalds and Burns.* By William M. Fahnestock, m.d.—Numerous as are the remedies recommended for scalds and burns, we cannot prevail upon ourselves to withhold adding another to the catalogue, which has proved highly serviceable in many cases under our direction; two of which were so extensive and severe as to deserve to be recorded, and may serve to illustrate the efficacy of the remedy.

The first was my brother, a child of four years of age, who fell into a tub of brine, which had just been taken from the kettle; he was scalded from head to foot. Having the tincture at hand, we had it immediately dashed over his face, and, as soon as his clothes could be removed, he was kept constantly wet with it. The convulsive symptoms which followed the accident were soon subdued, and so grateful was the application, that the first words he uttered were "pour more on—pour more on." In the course of ten or fifteen minutes all the pain and distress was assuaged, and he sunk into a sound sleep, from which he awoke and recovered, with scarcely any inconvenience but slight abrasion of the skin, at places where it became corrugated on the parts on which he rested during the application of the remedy.

The second, W. F. R., an infant of four months of age, who, while having his clothes changed, and being entirely naked, had the boiling drippings of a frying pan turned upon him. The chest, abdomen, genitals, nates, and thighs, were deluged with the scalding fat. Being present, an inmate in the house, we had the fat wiped off in an instant and applied the tincture. After continuing to bathe him ten or fifteen minutes, all the unpleasant symptoms subsided, and he became comparatively quite at ease.

Scarcely any redness remained on the scalded surface in either of the above cases, or any thing left to attract attention, but the yellow discolouration of the skin from the tincture. There may be some difficulty in accounting for the precise modus operandi of this article. Whether it be simple evaporation of the alcoholic preparation abstracting heat, an anodyne power suspending nervous exaltation, or a direct revulsion, we have not space, at present, to discuss.

How far the medication may be useful or proper, further experiments must determine. If it act entirely on an anodyne impression, its efficacy may be limited to the early stage of the accident, as in the above cases, before re-action had much advanced. Under this view, the modus operandi may readily be explained; by allaying nervous exaltation, and consequently preventing vascular influx and congestion. If it act by evaporation, the surface had better be exposed, but we have derived more advantage by having the injured part protected by cotton steeped in the tincture. Whether it act as a refrigerant, sedative, or stimulant, we deem it much preferable to the Kentish liniment, or the spirit of turpentine, as it may be applied with much more facility, and without any apprehension of irritating the adjacent parts. —*American Journal of Med. Sciences.*

---

### MATERIA MEDICA.

*Arsenic in large Doses.* —We have received a communication from R. Darkin, m.d. of Columbus, N. J. in which he states, that he has employed, at the suggestion of Dr. Budd, of Mount Holly, N. J. arsenic in large doses, as a remedy for intermittent fever, and with great success. He gives it in the form of pill, in doses of one-fourth of a grain, four times a day; in one case he says he gave as much as five grains in three days. He says that he has never seen any serious injury result from these large doses.—*Ibid.*

---

### NATURAL HISTORY.

*Fertility of the Unio Pictonum.* Dr. Unger, in his interesting anatomico-physiological account of this animal, published at Vienna in 1827, already mentioned, reckoned, in a full-grown animal, 300,000 embryos and young individuals. This extraordinary abundance, which does not occur in animals lower in the scale, as in the medusæ, appears even considerable when contrasted with the fecundity of insects. The vast number is probably the production of only a single year, which will give for the whole life of the animal a produce of many millions of individuals.

---

MISCELLANEOUS.

*Connexion of Diseases with the Rock Formations of a Country.* Amongst a great many of the communes of Calvados, in France, near to each other, and exposed to the same climatic influences, there is one which is particularly liable to fever. Nearly the whole of these communes are situated upon lias and red marl, and some other clayey formations, which retain at the surface a humidity favorable for the formation of fogs. On the contrary, the communes situated on rocks having a loose texture, and which permit the rain-water to escape more easily, such as the great oolite, chalk, &c. or which do not present any beds capable of arresting the course of the water, as granite and certain slates, appear less liable to fevers. It results from these general considerations, that the soil, by its greater or less hygroscopic quality, may have an effect on the state of health, by favoring more or less the developement of certain diseases. M. de Caumont does not regard this observation as new, but communicates it with the view of ascertaining in what proportions (every thing being equal,) the fevers and other maladies are developed in the principal geological regions of Calvados; for example, in that of granite, slate, limestone, clay, &c.—*Journal de Geologie.*

